# International comparison of health spending and utilization among people with complex multimorbidity

**DOI:** 10.1111/1475-6773.13708

**Published:** 2021-08-05

**Authors:** Jose F. Figueroa, Irene Papanicolas, Kristen Riley, Olukorede Abiona, Mina Arvin, Femke Atsma, Enrique Bernal‐Delgado, Nicholas Bowden, Carl Rudolf Blankart, Sarah Deeny, Francisco Estupiñán‐Romero, Robin Gauld, Philip Haywood, Nils Janlov, Hannah Knight, Luca Lorenzoni, Alberto Marino, Zeynep Or, Anne Penneau, Kosta Shatrov, Onno van de Galien, Kees van Gool, Walter Wodchis, Ashish K. Jha

**Affiliations:** ^1^ Department of Health Policy and Management Harvard T.H. Chan School of Public Health Boston Massachusetts USA; ^2^ Department of Health Policy London School of Economics London UK; ^3^ Centre for Health Economics Research and Evaluation (CHERE) University of Technology Sydney New South Wales Australia; ^4^ Radboud University Medical Center Radboud Institute for Health Sciences, Scientific Center for Quality of Healthcare Nijmegen The Netherlands; ^5^ Institute for Health Sciences in Aragon (IACS) Zaragoza Spain; ^6^ Dunedin School of Medicine University of Otago Dunedin New Zealand; ^7^ KPM Center for Public Management University of Bern Bern Switzerland; ^8^ Hamburg Center for Health Economics Universität Hamburg Hamburg Germany; ^9^ The Health Foundation London UK; ^10^ Otago Business School University of Otago Dunedin New Zealand; ^11^ The Swedish Agency for Health and Care Services Analysis Stockholm Sweden; ^12^ Health Division Organisation for Economic Co‐operation and Development (OECD) Paris France; ^13^ Institute for Research and Documentation in Health Economics (IRDES) Paris France; ^14^ Zilveren Kruis Leusden The Netherlands; ^15^ Institute of Health Policy Management & Evaluation University of Toronto Toronto Ontario Canada; ^16^ Brown School of Public Health Providence Rhode Island USA

**Keywords:** diabetes, health care spending, heart failure, high‐cost patients, high need, international comparison

## Abstract

**Objective:**

The objective of this study was to explore cross‐country differences in spending and utilization across different domains of care for a multimorbid persona with heart failure and diabetes.

**Data Sources:**

We used individual‐level administrative claims or registry data from inpatient and outpatient health care sectors compiled by the International Collaborative on Costs, Outcomes, and Needs in Care (ICCONIC) across 11 countries: Australia, Canada, England, France, Germany, the Netherlands, New Zealand, Spain, Sweden, Switzerland, and the United States (US).

**Data Collection/Extraction Methods:**

Data collected by ICCONIC partners.

**Study Design:**

We retrospectively analyzed age–sex standardized utilization and spending of an older person (65–90 years) hospitalized with a heart failure exacerbation and a secondary diagnosis of diabetes across five domains of care: hospital care, primary care, outpatient specialty care, post–acute rehabilitative care, and outpatient drugs.

**Principal Findings:**

Sample sizes ranged from *n* = 1270 in Spain to *n* = 21,803 in the United States. Mean age (standard deviation [SD]) ranged from 76.2 (5.6) in the Netherlands to 80.3 (6.8) in Sweden. We observed substantial variation in spending and utilization across care settings. On average, England spent $10,956 per person in hospital care while the United States spent $30,877. The United States had a shorter length of stay over the year (18.9 days) compared to France (32.9) and Germany (33.4). The United States spent more days in facility‐based rehabilitative care than other countries. Australia spent $421 per person in primary care, while Spain (Aragon) spent $1557. The United States and Canada had proportionately more visits to specialist providers than primary care providers. Across almost all sectors, the United States spent more than other countries, suggesting higher prices per unit.

**Conclusion:**

Across 11 countries, there is substantial variation in health care spending and utilization for a complex multimorbid persona with heart failure and diabetes. Drivers of spending vary across countries, with the United States being the most expensive country due to high prices and higher use of facility‐based rehabilitative care.

**What is known on this topic**

- Health systems are structured and financed differently.
- Patients with complex multimorbidity are more susceptible to poor quality of care and incur higher health care costs than other patient groups.
- International comparisons of health systems mostly focus on the inpatient setting, with limited data evaluating differences in care across different components of the health care system.

**What this study adds**

- This study compares health care utilization and spending across 11 high‐income countries for an older person aged 65–90 years hospitalized with a heart failure exacerbation and a secondary diagnosis of diabetes, across five domains of care: acute hospital care, primary care, outpatient specialty care, post–acute rehabilitative care, and outpatient drugs.
- Compared to other countries, the United States incurred the highest average of health care spending per person due to high unit expenditures and higher use of facility‐based post–acute rehabilitative care.
- All countries spent a substantial amount for people with heart failure over the course of the year, which increases incrementally among individuals with more complex comorbidities.

## 1 INTRODUCTION

As populations across the globe continue to grow and age, health systems are faced with the challenge to manage the increasing prevalence and impact of multimorbidity in older adults of their populations. In 2016, the World Health Organization declared the rise in multimorbidity, defined as the co‐occurrence of at least two chronic conditions in a given individual, a worldwide epidemic.^1^

A recent National Academy of Medicine (NAM) report^2^ highlighted that among people with multimorbidity, those with major chronic conditions—including heart failure, diabetes, and chronic obstructive pulmonary disease (COPD)—should be considered a priority group by health systems as they are more susceptible to poor quality of care and incur higher health care costs for several reasons. First, these individuals are usually of advanced age with long‐term chronic conditions that compromise their functional and cognitive abilities, thus limiting their ability to care for themselves effectively.^1^, ^3^ Second, their treatment often includes complex management regimens, which leads to issues of polypharmacy, adverse drug events, and medication adherence.^1^, ^4^ Finally, these patients are also at a higher risk for suffering the consequences of fragmented care, such as lack of coordination and poor communication, due to their frequent and complex interactions with multiple health care providers.^5^, ^6^ Comparisons across health systems may offer new knowledge regarding cost and quality differences and create opportunities for improvement internationally. To date, we have limited evidence of cross‐country variability in the management of these complex populations and the extent to which systems make different, more efficient, use of certain care settings.

Therefore, as part of the International Collaborative on Costs, Outcomes, and Needs in Care (ICCONIC), we sought to understand whether there are key differences in health care spending and utilization among a persona with complex multimorbidity across 11 countries.^7^ The countries in this study include Australia, Canada, England, France, Germany, the Netherlands, New Zealand, Spain, Sweden, Switzerland, and the United States (US). Using the NAM high‐need, high‐cost framework, we identified a comparable persona, defined as an older person hospitalized with a heart failure exacerbation with a co‐occurrence of diabetes. We then evaluated patterns of resource use across countries for 365 days following a heart failure exacerbation.

Specifically, in this study, we asked the following key questions. (1) How does health care spending vary across different components of the care pathway—including primary care, outpatient specialty care, hospital care, drugs, and post–acute rehabilitative care—among 11 high‐income countries (where data are available)? (2) To what extent do these variations in health care spending persist after taking into account the relative country‐specific utilization across care settings? Finally, (3) among a more complex subset of patients hospitalized with heart failure with a comorbidity of diabetes, how does an additional comorbidity of COPD influence health care spending across countries relative to those without diabetes or COPD?

## 2 METHODS

### 2.1 Country datasets

The ICCONIC collaborative uses patient‐level data from 11 countries: Australia, Canada, England, France, Germany, the Netherlands, New Zealand, Spain, Sweden, Switzerland, and the United States. Specific details of each dataset used can be found in Appendix Table A1. The representativeness of each dataset is found in Appendix Table A2. The years used in this dataset were between 2015 and 2017 across most countries, except in England (2014–2016) and Australia (2012–2016), both of which used more years of data to obtain a larger sample size. Data in three countries—New Zealand, Sweden, and Switzerland—covered their entire population. Data in three other countries were regional samples—Australia (New South Wales), a full region in Canada (Ontario province), and Spain (Aragon region). Data in the remaining five countries were large, diverse national samples, including in England, France, Germany, the Netherlands, and the United States. The proportion of patients covered in each dataset varied across countries, from 3% in Spain (Aragon) to 100% in New Zealand, Sweden, and Switzerland.

Datasets included patient‐level linked data across different domains of care, including primary care, outpatient specialty care, acute hospital care, post–acute rehabilitative care, and outpatient pharmaceuticals. Countries' ability to collect comprehensive data across each domain for health care utilization and spending categories varied (see Appendix Tables A3 and A4 for details).

### 2.2 Sample selection of heart failure persona

Using the high‐need, high‐cost framework identified by NAM, we selected a patient persona that is representative of an “older person with complex multimorbidity.” This group of patients was identified by NAM as a population thought to especially benefit from improving the value of care and reducing inefficiencies in care provision. The NAM framework was constructed by a team of clinicians, academics, economists, and policy makers.^2^

To identify a specific persona that is reflective of the older person with complex multimorbidity, we used a consensus decision making process among members of the ICCONIC collaborative, which included physicians, policy makers, data scientists, statisticians, and health economists. We defined the patient vignette with specified demographic and clinical criteria that were common enough across countries to allow for adequate sample selection. We then built a construct that reflected these requirements, which were identified as an older person, aged 65–90 years old, who was hospitalized with a heart failure exacerbation and who had a comorbidity of diabetes at the time of hospitalization. As a sub‐sample, we also identified patients who had an additional comorbidity of COPD in a subset of countries that were able to do so.

We required at least 2 years of data. The first identification step was to identify all patients in year 1 who were hospitalized with a primary diagnosis of congestive heart failure with International Classification Code‐10 of I50.x, as defined by the World Health Organization. Patients were included in the sample only once from their first recorded hospitalization in year 1. Given the lack of comprehensive longitudinal data across most countries, we were unable to know if the hospitalization was the first hospitalization related to heart failure or not. We then identified the subset of patients who at the time of admission also had a diagnosis of diabetes, including International Classification of Diseases, Tenth Revision (ICD‐10) codes: E11.x, E12.x, E13.x, and E14.x. Of note, type 1 diabetes (E10.x) was excluded from these analyses a priori in order to identify a more uniform population across countries. Finally, we identified subsets of patients that had an additional comorbidity of COPD, including those with ICD‐10 codes: J41.x, J42.x, J43.x, and J44.x. We also identified the prevalence of other chronic conditions using Elixhauser comorbidity definitions.^8^ All countries used ICD‐10 classification codes, except Spain (which used ICD‐9 codes) and the Netherlands (which used a set of codes given by the data supplier that matched the ICD‐10 diagnostic codes).

### 2.3 Defining spending and utilization categories

Across countries, we tracked spending and utilization across five domains of care: (1) acute hospital care, (2) post–acute rehabilitative care, (3) primary care, (4) outpatient/ambulatory specialty care, and (5) outpatient pharmaceuticals. For definitions of spending categories and utilization measures, please see Appendix Tables A5 and A6, respectively.

**FIGURE A1:**
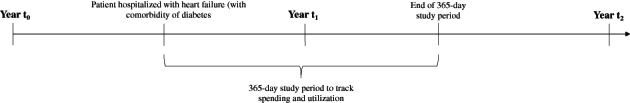
Identification strategy for persona with heart failure and diabetes

We then started from day 1 of hospitalization and followed patients across the five domains, where data were available across countries, for the 365 days that followed or until date of death if the patient did not survive the full year (see Appendix Figure A1).

### 2.4 Analysis

Due to constraints in data sharing, each country was only able to provide aggregated data for comparison. For each of the utilization and spending categories, countries supplied aggregated data reflecting mean use and spending in five age groups (65–69, 70–74, 75–79, 80–84, and 85–90 years—last age cohort consisted of 6 years) stratified by sex. While all countries have expenditure data, the cost accounting methods used to estimate expenditure differ across countries, in part due to the differences in payment systems adopted (Appendix Table A7). For example, some countries are able to report direct spending from incurred costs (those with full costing systems) while others provide information on reimbursement for specific episodes (e.g., diagnosis‐related groups) or an unweighted average unit prices. There are also differences in payment systems within countries across the different sectors. Finally, the reporting and imputation of capital investments or indirect costs also varies by system.

In order to reliably compare spending, we applied the Organization for Economic Cooperation and Development's (OECD) Actual Individual Consumption Purchasing Power Parities (AIC PPPs) to the expenditure data. AIC PPPs, rather than Gross Domestic Product Purchasing Power Parities (GDP PPPs), are currently used by the OECD as the most reliable economy‐wide conversion rates for health expenditure. Across each country, we applied 2017 AIC PPPs to all expenditures by the 5‐year age groups stratified by sex. In work by Lorenzoni et al., we further examine differences in spending when adjusting for pathway‐specific PPPs, using data from the collaborative, and also health‐specific PPPs.

We then performed an age and sex direct standardization using the US sample population as the reference population for all countries. For each age group and sex, all utilization and spending measures were weighted and recalculated against the US sample population weights. The totals were then calculated by weighting each individual group and sex's shares on the original country‐specific total to generate total, male, and female age–sex standardized values. We then compared age–sex standardized results across each category of spending and utilization. Our primary persona was the person hospitalized with heart failure and with a concomitant diagnosis of diabetes. Across a subset of countries with complete data, we also performed an additional comparison that compared the relative total spending across the five domains of care of patients hospitalized with heart failure who did not have a diagnosis of COPD or diabetes with the primary persona (those with a secondary diagnosis of diabetes) and a more complex persona (those secondary diagnoses of both diabetes and COPD).

### 2.5 Ethics approval

The Institutional Review Board at the Harvard T.H. Chan School of Public Health approved this study. In Spain, participation in this project was approved by Ethics Committee for Clinical Research in Aragon. In Germany, ethical clearance was provided by the ethical review board of the Faculty of Business, Economics and Social Sciences of Universität Hamburg. In Switzerland, ethical clearance was provided by the ethical review board of the Faculty of Business, Economics and Social Sciences of University of Bern. In Canada, participation in this project was authorized under section 45 of Ontario's Personal Health Information Protection Act (PHIPA) and does not require review by a research ethics board.

## 3 RESULTS

### 3.1 Sample characteristics

Across countries, we identified the following number of patients hospitalized with heart failure with a secondary diagnosis of diabetes: 3014 patients in Australia (New South Wales); 6305 patients in Canada (Ontario); 742 patients in England; 21,957 patients in France; 10,583 patients in Germany; 2035 patients in the Netherlands; 1572 patients in New Zealand; 1270 patients in Spain (Aragon); 4615 patients in Sweden; 3369 patients in Switzerland; and 21,803 patients in the United States (Table 1). The mean age ranged from 76.2 (5.6) in the Netherlands to 80.3 (6.8) in Sweden. The proportion of women was as low as 36.5% in Australia to as high as 50.7% in Germany.

**TABLE 1:** Sample characteristics of patients hospitalized with heart failure with a secondary diagnosis of diabetes across countries

	Australia (New South Wales)	Canada (Ontario)	England	France	Germany	The Netherlands	New Zealand	Spain (Aragon)	Sweden	Switzerland	United States
Sample size	3014	6305	742	21,957	10,583	2035	1572	1270	4615	3369	21,803
Age											
Mean (SD)	79.4 (6.8)	78.1 (7.0)	78.7 (6.6)	79.1 (6.9)	79.0 (6.4)	76.2 (5.6)	77.3 (6.9)	80.2 (5.1)	80.3 (6.8)	78.6 (6.5)	77.2 (7)
Median	80	79	79	80	79	77	77	81	81	77	77
Proportion female	36.5%	45.4%	42.5%	46.0%	50.7%	48.4%	42.4%	46.9%	41.3%	42.6%	50.0%
Comorbidities captured in dataset											
Mean (SD)	5.9 (2.3)	3.5 (1.3)	5.1 (1.5)	5.5 (1.9)	6.1 (2.0)	n/a	3.9 (1.4)	5.6 (2.1)	3.2 (1.2)	5.9 (1.7)	6.3 (1.7)
Median	6	3	5	5	6	n/a	4	5	3	6	6
Chronic conditions											
Heart failure	100%	100%	100%	100%	100%	100%	100%	100%	100%	100%	100%
Diabetes	100%	100%	100%	100%	100%	100%	100%	100%	100%	100%	100%
Depression	4.0%	1.5%	3.5%	4.7%	6.3%	n/a	0.4%	6.6%	1.0%	8.0%	1.5%
Hypertension	51.6%	41.1%	64.2%	69.0%	82.4%	n/a	51.2%	75.7%	57.2%	81.2%	89.3%
Renal failure	49.3%	5.5%	39.2%	34.9%	59.0%	n/a	47.3%	42.9%	25.8%	62.1%	54.5%
Chronic obstructive pulmonary disease	26.2%	16.3%	31.0%	17.1%	22.7%	n/a	10.3%	24.6%	18.7%	17.7%	42.7%

*Note*: The Netherlands was unable to calculate prevalence of each comorbidity. The data supplier gave our research collaborator access only to necessary information for this study, which only included relevant diagnoses to participate in this study.

Countries varied in the ability to capture secondary diagnoses in the index hospitalization. The United States (6.3, SD 1.7) and Germany (6.1, SD 2.0) had the highest mean number of comorbidities captured while Canada (3.5, SD 1.3) and New Zealand (3.9, SD 1.4) captured the lowest number in their datasets. The ability to track diagnosis varied significantly based on the financial incentives to upcode diagnosis and the number of secondary diagnoses that are recorded in each dataset.^7^, ^9^

### 3.2 Differences in spending across countries

**FIGURE 1:**
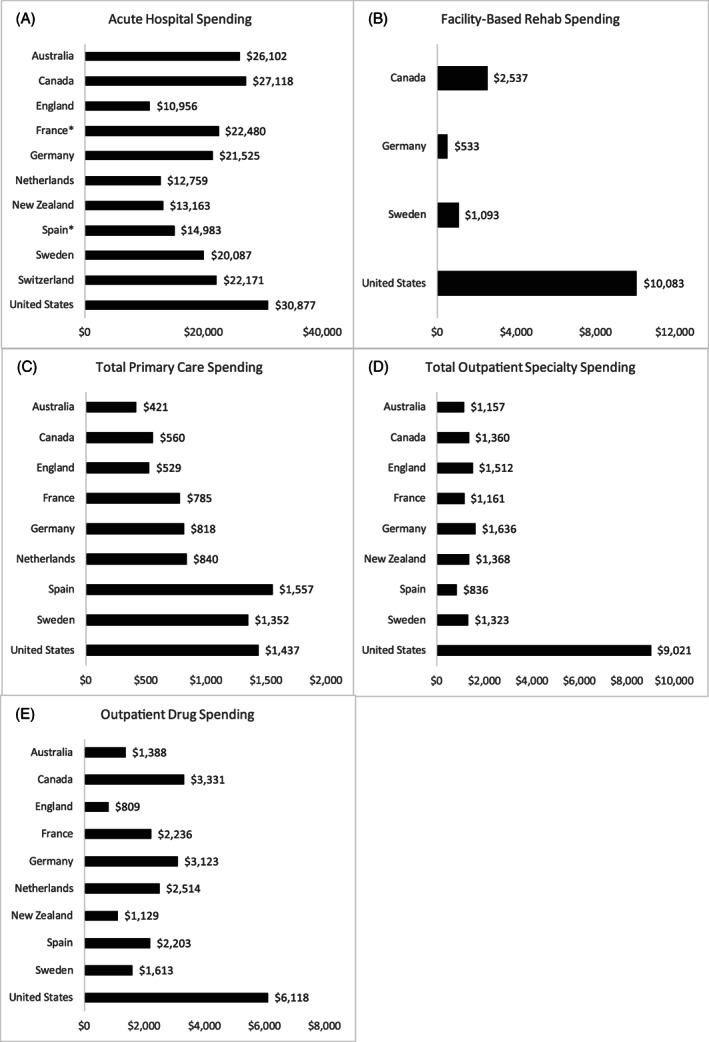
Differences in health care spending by care domains over 365 days for persona hospitalized with heart failure with a comorbidity of diabetes

**FIGURE A2:**
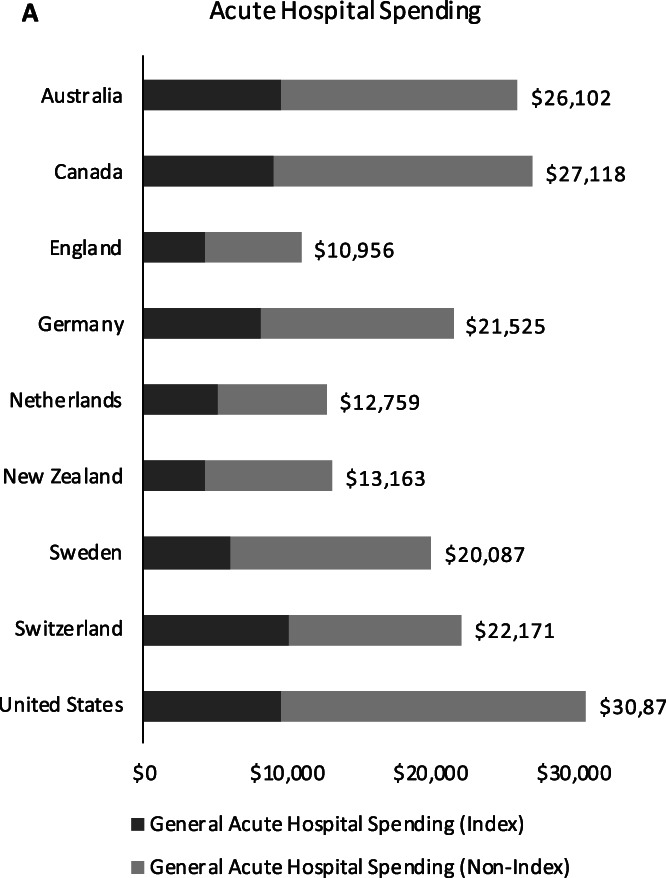
Differences in health care spending in the acute hospital setting related to the index admission and subsequent (nonindex) admissions

Figure 1 illustrates differences in age–sex standardized spending across five settings of the care pathway, including acute hospital care, facility‐based rehabilitative care, total primary care, total outpatient/ambulatory specialty care, and total outpatient drug spending. Across countries, there was wide variation in spending related to acute hospital and emergency care. There was almost a threefold difference in hospital spending between England ($10,956 per person) over the course of the year compared to the United States ($30,877 per person). The majority of spending in the acute hospital sector was related to spending in subsequent hospitalizations across all countries and not related to the index hospitalization (see Appendix Figure A2).

There were wide differences in spending related to primary care services, with as low as $421 per person in Australia to as high as $1557 per person in Spain. There was less variation across countries related to outpatient specialty care with one notable exception in the United States, which had significantly more spending at $9021 per person. Spending related to drugs was also the highest in the United States ($6118 per person), while it was the lowest in England ($809 per person).

### 3.3 Differences in utilization across countries

**FIGURE 2:**
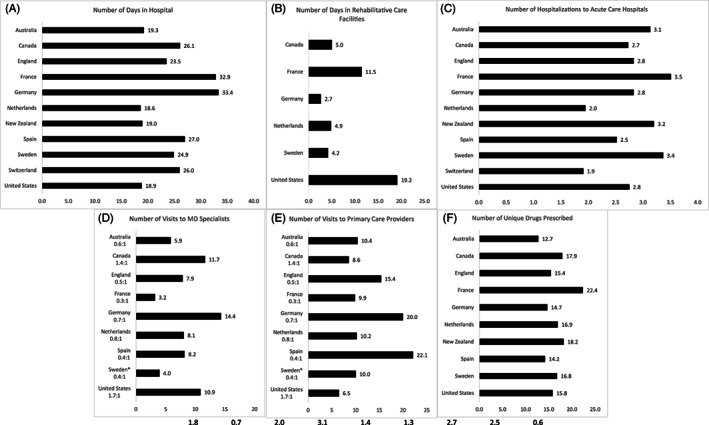
Differences in utilization of health care services over 365 days. Primary care visits for Sweden represent average yearly consumption for this cohort rather than linked patient‐level data

**FIGURE A3:**
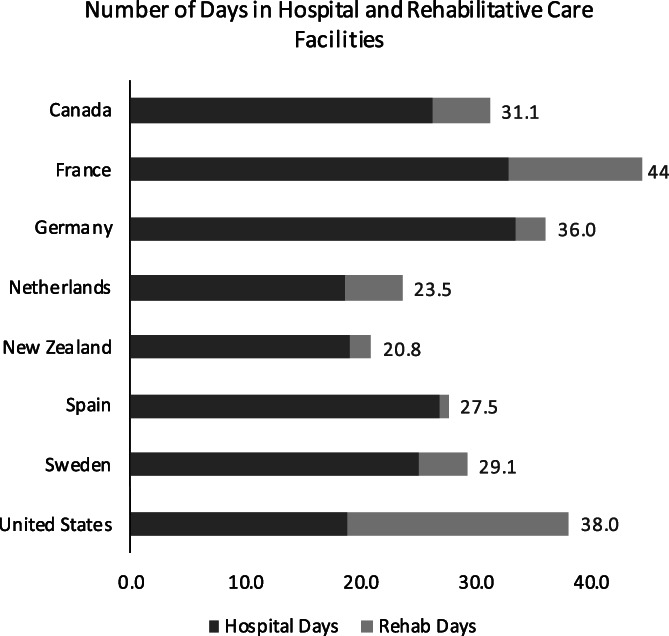
Mean number of combined days in hospital and rehabilitative care facilities over 365 days, age–sex standardized

Figure 2 illustrates differences in utilization of key health care services. Switzerland and the Netherlands had the fewest number of hospitalizations per person, while France and Sweden had the highest number of hospitalizations per person (Figure 2(C)). On average, the Netherlands (18.6 days) and the United States (18.9 days) had the lowest number of days in the hospital, while France (32.9 days) and Germany (33.4 days) had the highest number of days in the hospital (Figure 2(A)). Of the four countries that had data for facility‐based rehabilitative care sector, Germany (2.7 days) had the fewest number of days spent in this sector while the United States (19.2 days) had the highest (Figure 2(B)). When combined, France and the United States had the highest number of days in hospitals and rehab facilities with 44.3 days per person in France and 38.0 days per person in the United States (Appendix Figure A3).

**FIGURE A4:**
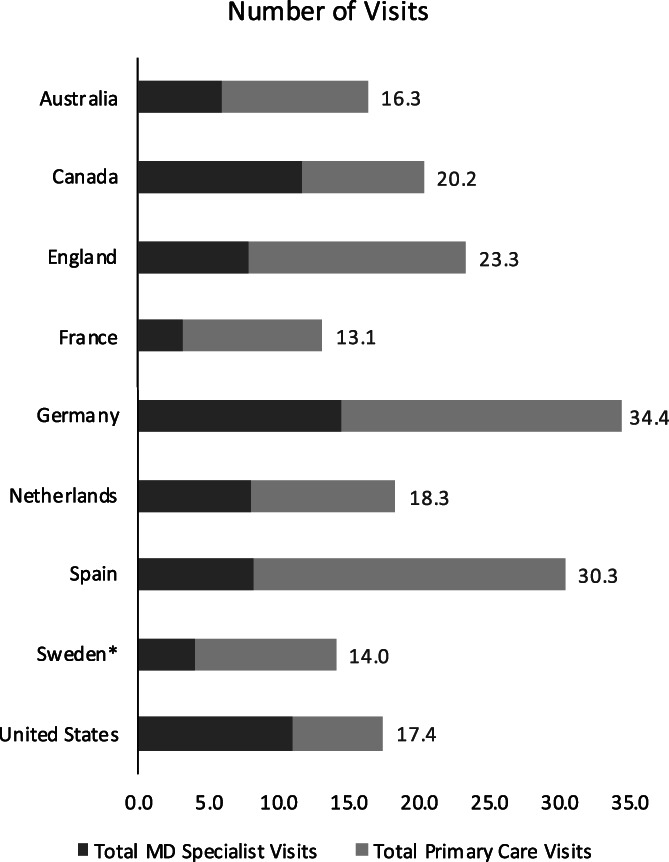
Mean number of combined visits to primary care providers or MD specialists over 365 days, age–sex standardized. MD: medical doctor. Primary care visits for Sweden represent average yearly consumption for this cohort rather than linked patient‐level data

The number of unique visits to primary care providers and outpatient specialists also varied substantially across countries (Figure 2(D),(E)). On average, the United States (6.5 visits) had the fewest number of visits to primary care providers while Spain (22.1 visits) and Germany (20.0 visits) had the highest. When combined, Germany and Spain had a higher number of visits across both specialists and primary care providers than other countries (Appendix Figure A4).

For unique drugs prescribed, Australia (12.7 drugs per person) had the fewest number of drugs prescribed, while France had the highest (22.4 drugs per person) (Figure 2(F)). The United States was an average utilizer at 15.8 drugs per person.

### 3.4 Spending per unit

**FIGURE 3:**
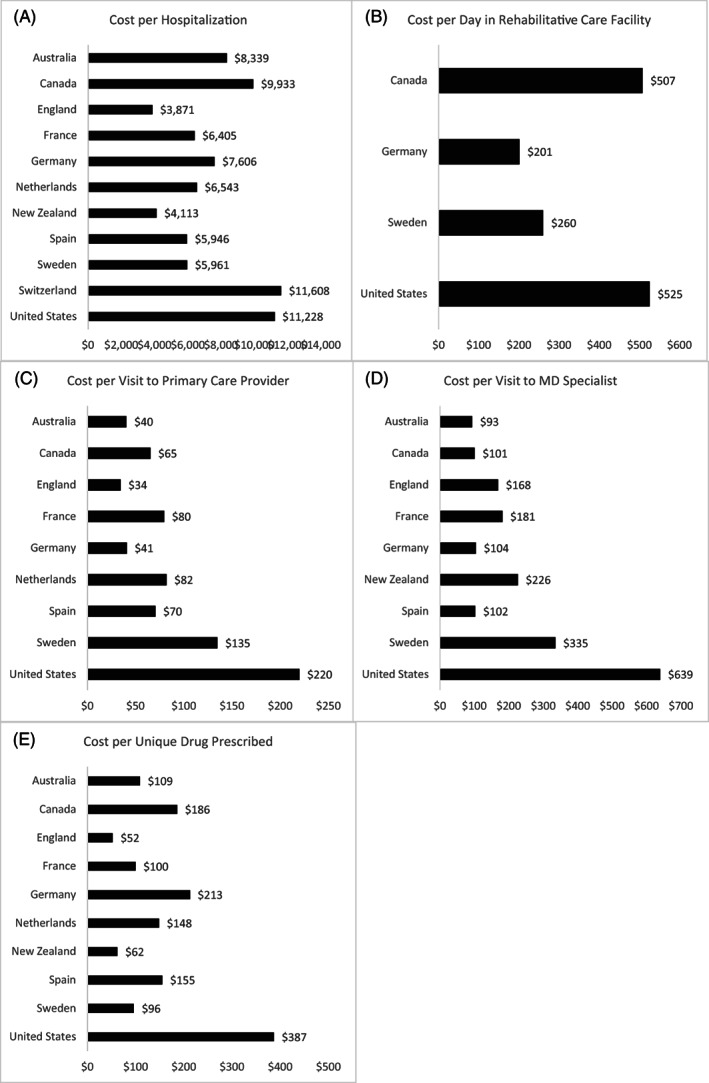
Utilization‐adjusted health care spending across countries over 365 days for heart failure persona with a comorbidity of diabetes

Across almost the different domains of care, the United States spent more per unit of health service across four sectors (primary care, specialty care, drugs, and post–acute rehabilitative care) than other countries (Figure 3). Only Switzerland spent slightly more per person in acute hospital care than the United States, which was a close second.

### 3.5 Relative spending increases by patient complexity

**FIGURE 4:**
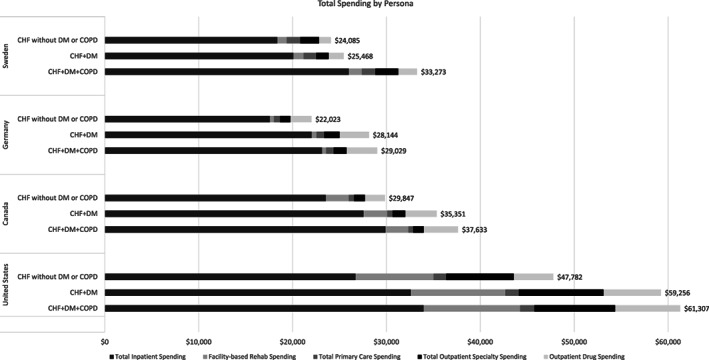
Differences in spending across different subtypes of heart failure patients with or without a comorbidity of diabetes and chronic obstructive pulmonary disease. Only Sweden, Germany, Canada, and the United States had comprehensive data on spending across all five categories. CHF, congestive heart failure; COPD, chronic obstructive pulmonary disease; DM, diabetes mellitus

**FIGURE A5:**
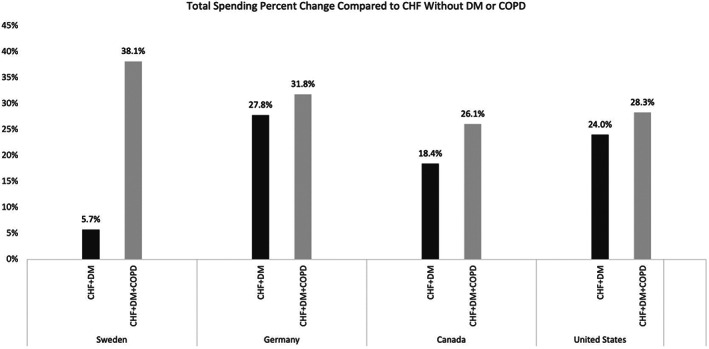
Percent change of yearly total spending for hospitalized heart failure patients with diabetes and chronic obstructive pulmonary disease relative to those without. CHF, congestive heart failure; COPD, chronic obstructive pulmonary disease; DM, diabetes mellitus

Among countries that had comprehensive data across all five care domains, as the complexity of the patient increased (those with diabetes and/or COPD relative to those without diabetes or COPD), the mean spending increased (Figure 4). The majority of the increase was related to increases in inpatient spending for heart failure patients with diabetes and COPD relative to those without these comorbidities. Across all four countries in this analysis, we observed increased levels of spending across all CHF personas as the level of complexity (additional comorbidities of diabetes and COPD) increased (Appendix Figure A5).

## 4 DISCUSSION

Across the globe, health systems are increasingly faced with the challenge of caring for older adults with complex multimorbidity. In this international comparison of 11 high‐income countries, we found substantial variation in both spending and utilization over the course of a year for a comparable multimorbid persona with heart failure and diabetes. All countries spent a substantial amount on this persona over the course of the year, which increases incrementally among individuals with an additional comorbidity of COPD.

Our findings shed valuable insights into what explains important differences in the care of this complex persona across countries. First, as prior work has documented, in the case of the United States, much of the increased spending is because “it's the prices, stupid.”^10^, ^11^ Across most health care sectors—including ambulatory specialty care, rehabilitative care, drugs, primary care, and hospital‐based care—the United States is paying substantially higher prices than other countries. Compared to England, for example, there is a threefold difference in spending in the acute care setting despite the United States having the same number of hospitalizations and a much lower length of stay. The United States also spends the highest per‐unit cost for each day of facility‐based rehabilitative care, visits to primary care doctors and outpatient specialists, and drugs.

Other important differences in variation of health care spending are related to differences in patterns of utilization of key services and where care provision occurs. For example, Canada, Germany, Spain, and Sweden all have longer lengths of stay in the hospital setting with fewer days in post–acute care rehabilitative facilities. In the United States, however, patients spend much less time in the hospital and, instead, are discharged quickly to the rehabilitative facilities (usually skilled nursing facilities). When combining hospital and rehabilitative days, the United States becomes the highest utilizer of care for this complex multimorbid persona. These results suggest a potential substitution effect from facility‐based rehabilitative care for hospital care.

In addition, these differences may in part be related to what each health system covers. For example, countries like the Netherlands, Spain, France, Sweden, and Canada, all have much more generous long‐term care coverage than the United States and England.^12^ In the United States, Medicare, which is the primary insurance for people aged 65 years and older, does not cover long‐term care services and support (unless dually enrolled and covered by Medicaid). In England, the National Health Services (NHS) does not cover long‐term care (also referred to as social care).^12^ Long‐term care is a separate government service provided at the local level and means tested.

Prior work has found that nearly 70% of adults who survive till age 65 years will require some form of long‐term care services and support.^13^ In countries like the United States, the majority of people rely on family and unpaid caregivers for this support.^13^ In other countries, however, long‐term care benefits exist and are covered by insurance, which includes caregiver support and nursing care at home. Some of this care is also likely shifted into the health care system, which takes the form of prolonged hospital days and prolonged courses of rehabilitative care in the United States. Our results suggest that policy makers in countries with limited long‐term care services, such as the United States, should consider strategies to improve access to affordable long‐term care services, especially given that caring for patients at hospitals and rehabilitative facilities is much more expensive than caring for them in residential settings.

Key cross‐country differences were also observed in the number and distribution of visits to specialty care versus primary care in the ambulatory setting. In countries like France, Germany, the Netherlands, Spain, and Sweden, we observed more visits for this persona with primary care providers than with specialists. Only Canada and the United States had a higher proportion of visits with specialists. Given that the price per specialist visit was much higher than the price per primary care visit across countries, higher spending in Canada and the United States is partially explained by more specialty care. It is important to note the fee‐for‐service Medicare program does not require a prior authorization or referral to see specialists. This is in contrast to countries such as England and New Zealand, which control utilization of specialty services by requiring referrals from primary care practitioners.^14^, ^15^ This is another area where there might be important differences in where similar types of care are received. For example, in England, the management of chronic patients occurs in the primary care setting, including onward community treatment and medication management. These activities may be occurring in specialist care in other systems such as Canada and the United States.^16^, ^17^, ^18^

### 4.1 Limitations

This study has notable limitations. First, there are important differences in country datasets used for this study. For example, some countries utilized administrative claims data from public or private insurers, while other countries used national registry data or large survey data that are linked with claims data. It is possible that the data captured in the regional databases may not be generalizable to other parts of the country. However, the use of a prespecified construct with clinical input from an advisory board was used to limit cross‐national differences and potential misclassifications. In the United States, data were limited to the Fee‐For‐Service Medicare population, and it did not include people enrolled in Medicare Advantage, the private option for beneficiaries. In addition, all countries had access to hospital data with diagnostic codes that allowed for the reliable identification of people with heart failure. Second, there are important differences in national coding practices between countries and cost‐accounting, which may influence the results. Where possible, we have documented these differences to identify potential sources of bias. Furthermore, many countries were missing key variables of utilization and spending, for example, those related to the post–acute rehabilitative care setting. Identification of specific procedures and tests in the hospital were also not able to be detected across countries due to data limitations. Long‐term data were also not available across the majority of countries, which may be important as well. However, we believe the dataset and comparison as currently collected offers important insights into the care for multimorbid personas across countries.

## 5 CONCLUSION

Across 11 high‐income countries, there is substantial variation in health care spending and utilization for a complex multimorbid persona with heart failure and diabetes. Drivers of spending vary across countries, with the United States being the most expensive country due to high prices and higher use of post–acute rehabilitative care.

## DISCLAIMERS

The results from New Zealand are not official statistics. They have been created for research purposes from the Integrated Data Infrastructure (IDI), which is carefully managed by Stats NZ. For more information about the IDI, please visit https://www.stats.govt.nz/integrated-data/.

The 45 and Up survey data used to represent Australia oversamples people older than age 80 and residents of rural and remote areas. The 45 and Up Study had a response rate of 18%, so the cohort might not be representative of the NSW population. Also, the survey focuses on NSW and may not be representative of the national sample for the same age group.

## APPENDIX A.

**TABLE A1:** Country datasets

Country	Datasets
Australia	Sax Institute's 45 and Up study.
Canada	Administrative claims data of the province of Ontario from the Ontario Ministry of Health and the Canadian Institute for Health Information through the Institute for Clinical Evaluative Sciences.
England	Primary care data from the Clinical Practice Research Datalink linked to secondary care data from Hospital Episode Statistics and Office for National Statistics death register.
France	Système National des Données de Santé/National Health Data System. ResidEhpad (long‐term care in residential facilities).
Germany	Administrative data of a large, nationally active health insurance with more than 8 m enrollees (BARMER) (includes utilization/costs of all sectors that are paid by health insurance).
The Netherlands	Zilveren Kruis insurance data (nationwide), which has about 30% of market share in the country.
New Zealand	The Integrated Data Infrastructure. The National Minimum Dataset (hospital admissions data). The pharmaceutical collection (medication dispensing data). The National Non‐Admitted Patient Collection (outpatient data).
Spain	Base de datos de usuario (National Health Service users dataset including insurees admin data). OMI‐AP (primary care electronic health records). Conjunto Mínimo Básico de Datos (administrative data for hospital discharges and outpatient contacts). Sistema de Información Hospitalaria (outpatient visits to specialized care). Receta Electrónica (e‐Prescription files). Facturación Recetas (billing files of over‐the‐counter prescriptions). Puesto Clínico Hospitalario de Urgencias (emergency care contacts).
Sweden	The national patient registry (inpatient and outpatient specialized care). The national prescription drug registry (outpatient pharmaceuticals). The national mortality registry. The national registry for interventions in municipal health care (enrollment in home medical care). The national registry of measures for the elderly and people with disabilities (long‐term care). Regional administrative registers of primary care consumption for the regions of Stockholm, Jönköping, Norrbotten, Skåne, and Västra Götaland.
Switzerland	Medical statistics dataset of the Federal Statistical Office (FSO), including hospital admissions records. Patient data from hospital‐based outpatient care dataset of the FSO. Short‐ and long‐term care facility records dataset of the FSO.
United States	Medicare fee‐for‐service data, 20% sample of all patients aged 65 years or older.

**TABLE A2:** Representativeness of Country Datasets

	Australia	Canada	England	France	Germany	The Netherlands	New Zealand	Spain	Sweden	Switzerland	United States
Year of data	2012–2016	2016–2017	2014–2017	2016–2017	2016–2017	2016–2017	2016–2017	2015–2016	2015–2016	2015–2016	2016–2017
Country population											
Total	23.5 million	35.2 million (Canada 2016)	55.3 million (in 2016)	64.5 million	82.2 million	16.9 million	4.7 million	46.6 million	9.9 million	8.4 million	323.1 million
Population age older than 65	3.5 million	5.9 million (Canada 2016)	9.9 million (in 2016)	12.3 million	16.8 million	2.4 million	733,272	8.3 million	1.9 million	1.5 million	49.2 million
Sample population (dataset)											
Representativeness of dataset	Regional data (New South Wales)	Regional Data (Ontario Province)	National sample	12 regions of France	National sample	National sample	Full national data	Regional Data (Aragon, Spain)	Full national data	Full national data	National sample
Total sample in dataset	267,086	14.5 million	7% of the UK population, representative in terms of age and sex*	43.1 million	8.6 million	30% market share*	4.7 million	1.3 million	9.9 million	8.4 million	6.4 million
Population age older than 65 in dataset	123,625	2.5 million		8.2 million	2.5 million		733,272	288,738 (21.73%)	1.9 million	1.5 million	5.4 million

*Note*: Specific full denominator was not able to be shared by the data supplier for the Netherlands.

* Specific full denominator could not be shared by the data provider.

**TABLE A3:** Data used to calculate specific health care utilization measures by country

	US	CA	DE	FR	ES	UK	AU	NZ	NL	CH	SE
*Utilization*											
Inpatient/acute care											
General acute hospitalizations	X	X	X	X	X	X	X	X	X	X	X
Days in hospital	X	X	X	X	X	X	X	X	X	X	X
Days in hospital (index)	X	X	X	X		X	X	X	X	X	X
Post–acute care rehab											
Days in rehabilitative care facility	X	X	X	X					X		X
Days in rehabilitative care facility (index)	X	X	X	X					X		
Primary care											
Primary care MD visits (or equivalent RNs/NPs)	X	X	X	X	X	X	X		X		X
Outpatient specialty care											
MD specialist visits	X	X	X	X	X	X	X	X	X		X
Drugs											
Number of unique drugs prescribed	X	X	X	X	X	X	X	X	X		X

*Note*: The two‐letter abbreviations represent the following countries: United States (US), Canada (CA), Germany (DE), France (FR), Spain (ES), England (UK), Australia (AU), New Zealand (NZ), the Netherlands (NL), Switzerland (CH), and Sweden (SE); the health care provider abbreviations used include, medical doctor (MD), registered nurse (RN), and nurse practitioner (NP).

**TABLE A4:** Data used to calculate health care spending across different domains of care by country

	US	CA	DE	FR	ES	UK	AU	NZ	NL	CH	SE
*Spending*											
Inpatient/acute care											
Total inpatient/acute hospital spending	X	X	X		X	X	X	X	X	X	X
Post–acute care rehab											
Facility‐based rehabilitative care spending	X	X	X		X						X
Facility‐based rehabilitative care spending (index)	X	X	X		X						
Home‐based rehabilitative care spending	X	X	X	X					X		
Primary care											
Total primary care spending	X	X	X	X	X	X	X		X		X
Primary care services spending	X	X	X	X	X	X	X		X		X
Outpatient specialty care											
Total outpatient specialty care spending	X	X	X	X	X	X	X	X		X	
Drugs											
Outpatient drug spending	X	X	X	X	X	X	X	X	X		X

*Note*: The two‐letter abbreviations represent the following countries: United States (US), Canada (CA), Germany (DE), France (FR), Spain (ES), England (UK), Australia (AU), New Zealand (NZ), the Netherlands (NL), Switzerland (CH), and Sweden (SE).

**TABLE A5:** Definitions of health care spending categories

Category	Definition
Inpatient and emergency care	
Acute care hospital spending	Acute hospitalizations are defined as any hospitalization that occurred in general hospitals for any condition. This includes physician fees, inpatient laboratory, imaging, and drugs given. All admissions were counted even if the patients were discharged the same day. It includes services provided in the emergency department.
Acute care hospital spending for index hospitalization	Same as above but for index hospitalization only.
Post–acute rehabilitative care	
Facility‐based post–acute rehabilitative care	All spending related to inpatient rehabilitative care or skilled nursing facilities or other spending related to rehabilitative care that requires a facility‐based stay
Facility‐based post–acute rehabilitative care for index hospitalization	Same as above but for rehabilitation following the index hospitalization.
Primary care	
Total primary care spending	Sum of spending categories below.
Primary care services provided by a MD	Costs for any service provided by general practitioners, general internists, or equivalent in a primary care/ambulatory setting.
Non‐MD primary care services	Primary care services provided by nurses, nurse practitioners, or other non‐MD equivalent (physician assistants, nurse practitioner). (Exclude phone calls)
Outpatient/ambulatory specialty care	
Total outpatient specialty care	Sum of spending categories below.
Outpatient MD specialty services	All visits to specialists who are MDs. These include MDs like cardiologists, gastroenterologists, surgeons, and so forth. Do not include radiologists or pathologists in this category.
Outpatient diagnostic MD specialist visits	All visits to radiologists or pathologists if it is an actual patient encounter.
Drugs	
Outpatient drugs	Any costs attributable to drugs prescribed to the patient in the outpatient setting are included in this section. Drugs administered as part of a hospital stay are not included in this category.
	Of note, drugs administered in the inpatient setting are included as part of inpatient/hospital costs above.

Abbreviation: MD, medical doctor.

**TABLE A6:** Definitions of health care utilization measures

Category	Definition
Inpatient and emergency care	
Hospitalizations to general acute care hospitals	All hospitalizations in general acute care hospitals. These include all admissions regardless of diagnosis (medical, surgical, and psychiatric conditions).
Days in hospital	Number of calendar days spent in the hospital. Day 1 is counted as day of admission.
Days in index hospitalization	Number of calendar days spent during the index hospitalization. Of note, if someone is transferred, please combine days from each hospital (refer to chart for details).
Post–acute care rehab	
Days in rehabilitative care facility	The number of days in a rehab facility, which includes inpatient rehabilitative care, skilled nursing facilities, or other facilities that provide rehabilitative care.
Days in rehabilitative care facility following index hospitalization	The number of days in a rehab facility following index hospitalization.
Primary care	
Total primary care visits	Sum of below categories
Primary care MD visits	All visits to primary care MDs (or equivalent). Please do not include telephone calls in this category since we are not able to capture this across most countries.
Primary care visits to nurses or equivalent	All primary care visits to nurses, nurse practitioners, or equivalent
Outpatient specialty	
MD specialist visits	All visits to specialists who are MDs. These include MDs like cardiologists, gastroenterologists, surgeons,and so forth. Do not include radiologists or pathologists in this category.
Diagnostic MD specialist visits	All visits to radiologists or pathologists if it is an actual patient encounter.
Drugs	
Number of unique drugs prescribed	The number of unique chemical compounds prescribed over the study year is counted here. This includes outpatient drugs only.

Abbreviation: MD, medical doctor.

**TABLE A7:** Health system characteristics by country^19^, ^20^

Health system characteristics	Australia	Canada	England	France	Germany	The Netherlands	New Zealand	Spain	Sweden	Switzerland	United States
Health system expenditure per capita (2019)	$5187	$5418	$4653	$5376	$6646	$5765	$4204	$3616	$5782	$7732	$11,072
Long‐term care expenditure per capita (2017)	$101	$923	$726	$780	$1099	$1385	‐	$317	$1425	$1409	$512
Health system expenditure % of GDP (2019)	9.3%	10.8%	10.3%	11.2%	11.7%	10.0%	9.3%	9.0%	10.9%	12.1%	17.0%
Long‐term care expenditure % of GDP (2017)	0.2%	1.9%	1.8%	1.8%	2.1%	2.6%	‐	0.9%	2.9%	2.4%	0.9%
Type of system (NHS type, private insurance, social insurance)	National public health insurance	National public insurance	National health care system (NHS)	Statutory insurance through employment‐based funds, tax‐financed coverage for unemployed	Mostly statutory insurance with some private insurance	Statutory, mandatory insurance through 11 private nonprofit carriers	National health care system	National health care system	National health care system with decentralized service delivery	National health insurance for basic coverage with optional supplementary insurance plans	Mix of public and private insurance
Population coverage (%)	100	100	100	100	100	100	100	100	100	100	91.5
Payment system of hospitals	Public hospitals mostly activity‐based (DRG) payments, with the rest global budgets while private hospitals mainly FFS	Global budgets, some case‐based payment	Mostly case‐based payments, with some local bundled‐payment pilots	Mostly case‐based (DRG) payments	Case‐based (DRG) payments	Case‐based (DRG) payments with a global budget	Case‐based payments	Mostly global budgets, some episode‐based payments	Mostly global budgets, remainder case‐based (DRG) payments or PFP	Case‐based (DRG) payments for inpatient care, FFS for outpatient care	Mix of FFS, case‐based (DRG), and per‐diem payments
Payment system of primary care (FFS, capitation, PFP, hybrid)	Mostly FFS, some PFP	Mostly FFS, some alternative payments or salaries	Mix of capitation, FFS, PFP	Mix of FFS and PFP, capitated annual bonus for chronic diseases	FFS	Mix of capitation and FFS, some bundled payments and PFP	Capitation and FFS, some incentive payments	Global budgets, capitation, PFP	Mostly capitation, some FFS or PFP	Mostly FFS, some capitation	Mostly FFS, some capitation and incentive payments

Abbreviations: DRG, diagnosis‐related group; FFS, fee‐for‐service; GDP, gross domestic product; NHS, National Health Service; PFP, pay for performance.
